# Event and Comment

**Published:** 1917-09

**Authors:** 


### DENTAL REGISTER

Vol. LXXI SEPTEMBER, 1917 No. 9

### EVENT AND COMMENT

SHOULD UNDERGRADUATE DENTAL STUDENTS ENTER THE ARMY? Ever since the entrance of this country in the European war there has been a very positive conviction in the medical and dental professions that this country should avoid the mistake made by other countries of sending their medical and dental men into the firing line, where they would be either killed or wounded so badly as to unfit them for either army or civil service. In this way England and France have suffered such losses of professional men as to seriously handicap the army service and to render it almost impossible for the people at home to have such medical and dental service as is needed to keep them in a moderate degree of good health and efficiency. In another place in this issue will be found a statement of what is happening because of the draft which is taking by the first call nearly one-fourth of all the undergraduate dental students. The significance of this will be better appreciated if we stop to consider that if the same proportions are kept up and the war should demand further calls, we shall have but a few dental students left to take the places made vacant in the army dental corps by accidents in the service, and of the civil practitioners who have enlisted or been drafted. We are making every effort to conserve our food and other war materials, and the query is, why can't we see far enough to know that we shall need every dentist we can get in the next few years, and make a proper effort to secure an adequate supply. The medical profession has taken warning from the fate of other countries and secured a ruling from the War Department that puts drafted undergraduate medical students on the enlisted reserve corps, and permits them to continue their studies until graduated, or until they are absolutely needed. The Secretary of War has declined to extend this privilege to dental students, on the ground that dentists are not necessary to the welfare of the army, and that it would be a class distinction to exempt them from line service. It seems strange that the army authorities can take such a position when they must know of the value of the dental service that has been rendered to the army and navy in the past; and also in view of the fact that because of the services rendered by the dental profession thousands of men have been made eligible for service under the present draft rules that otherwise could not have qualified. We have no intention of discouraging the efforts of our authorities to mobilize an ample army by any suggestions in this comment, but we believe it will be infinitely wiser to recruit our army from sources that can better be spared than to take out of training for a very much needed service, both in the army and at home, our dental students, especially those who will naturally be prepared for skillful and helpful service of a most desirable character practically as soon as they could be trained for line fighting. We don't believe the matter has been fully presented to the authorities or they would certainly take action similar to that already taken for engineering and medical students. It is a most important issue, and every member of the dental profession is competent to express an intelligent opinion on this matter, and he should take it upon himself to convey his earnest convictions to his representatives in Congress. We would not suggest this matter except that we have abundant evidence of the tremendous value of dentistry to the armies on the fighting front.

